# Cytotoxic Molecules as Potential Biomarkers for Active and Inactive Systemic Lupus Erythematosus

**DOI:** 10.3390/biomedicines13071559

**Published:** 2025-06-25

**Authors:** Paola Santana-Sánchez, Astrid Asminda Ramírez-Pérez, Paolo Alberti-Minutti, Julián A. Gajón, Laura C. Bonifaz, Norberto Sánchez-Escobar, María Victoria Legorreta-Haquet, Luis Chávez-Sánchez, Adriana Karina Chávez-Rueda

**Affiliations:** 1 Unidad de Investigación Médica en Inmunología, Unidad Médica de Alta Especialidad (UMAE) Hospital de Pediatría, Centro Médico Nacional Siglo XXI, Instituto Mexicano del Seguro Social, Mexico City 06720, Mexico; pao-ss@live.com.mx (P.S.-S.); norbertosanchezescobar@gmail.com (N.S.-E.); vileha14@yahoo.com.mx (M.V.L.-H.); luischz@yahoo.com (L.C.-S.); 2 Doctorado en Ciencias Biológicas y de la Salud, Universidad Autónoma Metropolitana, Mexico City 14387, Mexico; 3 Servicio de Reumatología, Unidad Médica de Alta Especialidad (UMAE) Hospital de Especialidades, Centro Médico Nacional Siglo XXI, Instituto Mexicano del Seguro Social, Mexico City 06720, Mexico; astrid.asminda@gmail.com; 4 Servicio de Medicina Interna, Unidad Médica de Alta Especialidad (UMAE) Hospital de Especialidades, Centro Médico Nacional Siglo XXI, Instituto Mexicano del Seguro Social, Mexico City 06720, Mexico; paolo.alberti@gmail.com; 5 Posgrado en Ciencias Bioquímicas, Universidad Nacional Autonoma de México, Mexico City 04510, Mexico; julian.argenis.23@gmail.com; 6 Unidad de Investigación Médica en Inmunoquímica, Unidad Médica de Alta Especialidad (UMAE) Hospital de Especialidades, Centro Médico Nacional Siglo XXI, Instituto Mexicano del Seguro Social, Mexico City 06720, Mexico; labonifaz@yahoo.com; 7 Coordinación de Investigación en Salud, Centro Médico Nacional Siglo XXI, Instituto Mexicano del Seguro Social, Mexico City 06720, Mexico

**Keywords:** systemic lupus erythematosus, SLEDAI 2-K, perforin, granulysin, granzyme-B, CD8^+^ T cell

## Abstract

**Objectives**: Systemic lupus erythematosus (SLE) is a chronic autoimmune disorder characterized by persistent inflammation. Reliable biomarkers for predicting disease reactivation are lacking. This study aimed to investigate serum cytokines and cytotoxic molecules in both the inactive (iSLE) and active (aSLE) phases to identify potential predictors of disease activity. **Methods**: Fifty-five SLE patients were classified as having iSLE (*n* = 36) or aSLE (*n* = 19) on the basis of clinical parameters and the Systemic Lupus Erythematosus Disease Activity Index 2000 (SLEDAI-2K). Serum levels of cytokines, cytotoxic molecules, and CD8^+^ cells were analyzed through flow cytometry and principal component analysis (PCA). Additionally, seventeen healthy donors (HDs) served as a control group. **Results:** Serum perforin (median: 2219 pg/mL; *p* = 0.0020) and granulysin (median: 1347 pg/mL; *p* = 0.010) levels were significantly higher in patients with aSLE than in patients with iSLE. In contrast, sFas levels were elevated in both SLE groups compared with those in the HD group. Moreover, increased perforin and granulysin levels were correlated with increased SLEDAI-2K scores, and the proportion of cytotoxic cells (CD8^+^granzyme-B^+^perforin^+^ cells) was correlated with disease activity. **Conclusions**: The increased levels of cytotoxic molecules and the high CD8^+^ cell proportions suggest that integrating these parameters with traditional biomarkers could enhance disease monitoring and management.

## 1. Introduction

Systemic lupus erythematosus (SLE) is a chronic autoimmune disorder characterized by the production of autoantibodies that target nuclear and cytoplasmic components, causing inflammation and tissue damage [1]. SLE presents diverse clinical manifestations, ranging from mild cutaneous involvement to severe organ dysfunction that affects the kidneys, lungs, cardiovascular system, central nervous system, and musculoskeletal system [2]. Although the exact etiology of SLE remains unclear, genetic predispositions and environmental triggers contribute to its ability to disrupt immune tolerance, resulting in hyperactivation of immune cells and chronic systemic inflammation [3].

Aberrant production and activity of cytokines and cytotoxic molecules have been proposed as key drivers of immune dysfunction in SLE, supported by both clinical and experimental studies, suggesting their involvement in immune cell dysfunction and tissue damage [4,5,6]. However, integrated studies that clearly delineate the mechanisms by which these mediators contribute to disease pathogenesis are still lacking. For instance, interleukin-6 (IL-6) and tumor necrosis factor-alpha (TNF-α) have been closely associated with SLE disease activity and the severity of organ involvement, highlighting their potential utility as biomarkers for disease progression [7,8,9].

Cytotoxic molecules, such as granzymes, perforin, and the Fas/FasL system, which are predominantly secreted by cytotoxic CD8^+^ T cells, are also emerging as critical regulators of immune-mediated damage and apoptosis in patients with SLE [10,11,12,13,14]. A combination of markers may offer a more comprehensive approach to understanding and predicting disease reactivation and progression. The growing evidence that links cytokines and cytotoxic molecules to SLE pathogenesis underscores their potential as valuable indicators of disease activity and long-term outcomes.

The heterogeneity of SLE complicates its diagnosis, prognosis, and treatment [15]. A key therapeutic goal is achieving clinical inactivity of SLE while managing accumulated damage [16]. Unpredictable flares and remissions, combined with the limited accuracy and reliability of current biomarkers, such as anti-dsDNA antibodies, highlight the need for reliable indicators of disease activity. Cytokines and cytotoxic molecules are particularly promising in this context, given their intimate associations with immune disturbances that drive SLE.

This study explored the use of cytotoxic molecules and cytokines as predictive biomarkers for SLE activity. Specifically, we sought to identify markers that could signal impending disease reactivation to enable earlier therapeutic interventions and reduce the risk of complications. By integrating multiple biomarkers into a panel, we propose a novel approach for predicting SLE outcomes and guiding personalized treatments.

## 2. Materials and Methods

### 2.1. Participant Recruitment

Fifty-five SLE patients were recruited from the rheumatology service at the Hospital de Especialidades CMN SXXI, IMSS (CDMX, Mexico) on the basis of the ACR (1997), SLICC, and EULAR/ACR classification criteria. Disease activity was assessed using the SLEDAI-2K score. Patients with a score ≥ 4 were classified as having active SLE (aSLE), whereas those with a score < 4 were considered to have inactive SLE (iSLE), in accordance with previously published studies [17,18]. Demographic data (sex, age, and age at diagnosis) were also collected. Only patients with complete clinical records were included.

The exclusion criteria included autoimmune diseases, infections, neoplasms, and recent changes in immunosuppressive therapy. The control group consisted of 17 healthy donors.

Written informed consent was obtained from all participants. The study followed the principles of the Declaration of Helsinki, Nuremberg Code, Finnish Treaty, Good Clinical Practice guidelines, and Mexico’s general health law for human research. The protocol was approved by the IMSS ethics committee (R-2022-785-047).

### 2.2. PBMC Collection

Peripheral blood mononuclear cells (PBMCs) were collected from all participants who consented to participate in the study. A total of 15 mL of peripheral blood was drawn into EDTA tubes. The blood samples were carefully layered onto Lymphoprep (Serumwerk, Bernburg, Germany) and centrifuged at 400× *g* for 30 min. The mononuclear cell layer, which contained lymphocytes and monocytes, was obtained.

### 2.3. Serum Collection

Peripheral blood was collected into tubes without anticoagulants. The serum was separated via centrifugation at 1000× *g*, aliquoted, and stored at −80 °C until further cytokine analysis.

### 2.4. Quantification of Molecules

The serum levels of 13 proteins were quantified using the LEGENDplex™ human CD8/NK panel (BioLegend, San Diego, CA, USA) following the manufacturer’s instructions. The proteins included IL-2, IL-4, IL-6, IL-10, IL-17A, IFN-γ, TNF-α, soluble Fas, soluble FasL, granzyme-A, granzyme-B, perforin, and granulysin. The samples were acquired on a MACSQuant analyzer 10 (Miltenyi Biotec, Bergisch Gladbach, Germany), and data were processed using the LEGENDplex™ data analysis software suite (BioLegend, San Diego, CA, USA; version available in 2024; https://legendplex.qognit.com/).

All measured values were included in the statistical analyses, including those near the lower limit of detection (LOD). No values were excluded as outliers or treated as undetectable. According to the manufacturer (LEGENDplex™ Data Analysis Software), the LODs for the analytes in serum ranged from 0.57 to 26.44 pg/mL (Supplementary Table S1). Although some measurements appeared close to zero in graphical representations, all values remained above the reported assay sensitivity threshold.

### 2.5. CD8^+^ T-Cell Staining

PBMCs were stained with anti-CD3 BV650 (OKT3) and anti-CD8 APC Fire 810 (SK1) antibodies for 20 min at 4 °C in the dark. After washing with FACS buffer, cells were permeabilized using the Transcription Factor Staining Buffer Set (BioLegend, USA) for 45 min at room temperature. Intracellular staining was then performed using anti-granzyme-B PerCP (QA18A28) and anti-perforin PerCP Cy5.5 (dG9) antibodies for 30 min.

Samples were acquired using a Cytek Aurora spectral flow cytometer (Cytek Biosciences, Fremont, CA, USA), and the data were analyzed with FlowJo v10 (Tree Star, San Carlos, CA, USA). The gating strategy included singlet discrimination, live/dead exclusion, and identification of CD3^+^CD8^+^ T cells. Fluorescence Minus One (FMO) controls were used to define positive gates for granzyme-B and perforin. All antibodies were purchased from BioLegend (USA).

### 2.6. Biomarker Association

Cytokine and cytotoxic molecule concentrations were quantified using flow cytometry and used to train a random forest (RF) algorithm to differentiate between the inactive (iSLE) and active (aSLE) SLE phenotypes. The analysis was performed in R Studio (v4.3.1), with the random forest package (v4.7-1.2), and 100 decision trees were utilized. Data normalization was not applied prior to model training, as random forest algorithms are generally robust to differences in variable scales. Feature importance was assessed by calculating the median value of the “Importance” metric across all trees in the model.

### 2.7. PCA-Based Clustering

Principal component analysis (PCA) was performed on the following eight variables: C4, IL-6, perforin, granulysin, granzyme-B, SLEDAI-2K score, CD8^+^, and CD8^+^ cytotoxic cells (granzyme-B^+^perforin^+^ cells). Prior to PCA, the data were standardized using Z-score normalization (centered to a mean of zero and scaled to unit variance) to ensure comparability and eliminate differences in scale. Data preprocessing and normalization were performed in RStudio (v4.3.1), and the factoextra package (v1.0.7) was used to visualize clustering patterns among the groups.

### 2.8. Multivariate ROC and Logistic Regression Analysis

To evaluate the discriminative potential of cytotoxic markers for classifying disease activity, we implemented a multivariate logistic regression model. The outcome variable was binary (aSLE vs. iSLE), and predictors included perforin, granulysin, granzyme-B, %CD8^+^, and %CD8^+^ cytotoxic cells. Model performance was assessed using receiver operating characteristic (ROC) curves, and the area under the curve (AUC) with 95% confidence intervals was calculated using the pROC package (v1.18.5) in RStudio (v4.3.1). Optimal cut-off points were determined using Youden’s index. Odds ratios (OR) and 95% confidence intervals were reported for each predictor.

### 2.9. Unsupervised Flow Cytometry Analysis

Live, single CD3^+^CD8^+^ T cells were concatenated and analyzed using the UMAP plugin (v4.1.1), with the parameters set to 15 nearest neighbors, a minimum distance of 0.5, and the Euclidean distance metric. Dimensionality reduction was followed by clustering using the FlowSOM plugin (v4.1.0, 8 clusters, 10 × 10 grid). Differentially enriched clusters between the iSLE and aSLE samples were identified using the T-Rex plugin (v1.2.2, K = 60, eps = 0.30). All unsupervised analyses were conducted in FlowJo v10.8.

### 2.10. Statistical Analysis

The data are presented as medians with interquartile ranges (IQR; Q1–Q3). Comparisons among the groups were performed using the Kruskal–Wallis test for nonparametric data, followed by Dunn’s multiple comparison post hoc test. Correlations between variables were analyzed using the nonparametric Spearman test. All statistical analyses were performed using GraphPad Prism software v8.0 (GraphPad Software, Inc., La Jolla, CA, USA). A *p*-value of <0.05 was considered statistically significant.

## 3. Results

### 3.1. Characteristics of the Participants

This study analyzed 55 samples from Mexican patients with SLE, including 36 patients in the iSLE phase and 19 patients in the aSLE phase. The demographic and clinical characteristics of this cohort are summarized in Table 1. The median (interquartile range) ages of the healthy donors (HDs), iSLE patients, and aSLE patients were 26 (25–53), 36 (30–44), and 34 (28–48) years, respectively. The median disease duration was longer in the aSLE patients (15 years) than in the iSLE patients (6 years). Information about the medications that the patients were taking at the time of sampling is also summarized in Table 1.

### 3.2. Global Differences in Cytokine and Cytotoxic Profiles in SLE

To explore overall immune dysregulation in systemic lupus erythematosus (SLE), we compared the serum levels of cytokines and cytotoxic molecules between healthy donors (HD) and the SLE group (combining inactive and active patients). The medians and interquartile ranges (Q1–Q3) for each biomarker are provided in Table 2.

Among the evaluated markers, TNF-α, sFas and sFas-L were significantly increased in the SLE patients compared to the healthy controls (*p* = 0.009, *p* < 0.001 and *p* = 0.045, respectively), indicating elevated systemic inflammation. Other markers, such as IFN-γ, granzyme-A, granzyme-B, and granulysin, showed a trend toward higher concentrations in the SLE group, although these did not reach statistical significance.

### 3.3. Different Serum Biomarkers Are Associated with the Phases of SLE

The serum levels of different biomarkers revealed distinct patterns between the iSLE and aSLE patients. We analyzed thirteen biomarkers, including cytokines and cytotoxic molecules. The levels of the cytokines IL-2, IL-4, IL-6, IL-10, IFN-γ, and IL-17A and those of the cytotoxic molecules granzyme-A and granzyme-B were generally low across both iSLE and aSLE groups, which was similar to the profiles observed in the HDs (Supplementary Figure S1).

**Figure 1:**
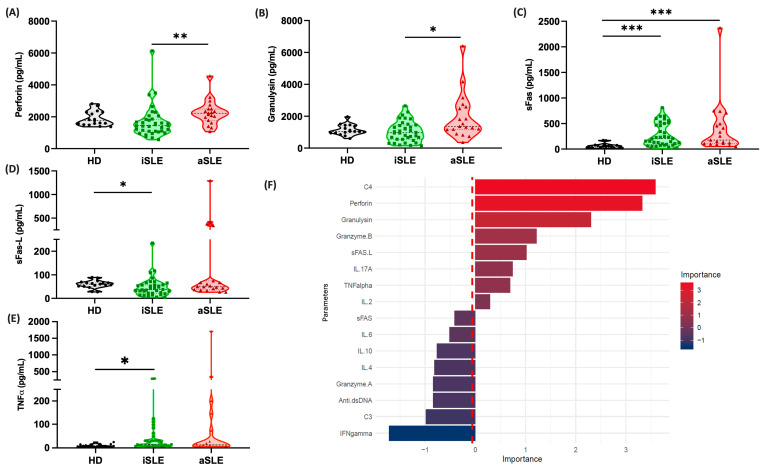
Biomarker profiles in SLE patients. Serum biomarkers were measured in 55 samples from SLE patients and analyzed in relation to disease activity during follow-up. (**A**–**E**) The levels of perforin, granulysin, sFas/sFas-L, and TNF-α (pg/mL) were compared among patients with healthy donors (HD; *n* = 17), inactive SLE (iSLE; *n* = 36) and active SLE (aSLE; *n* = 19) patients. The violin plots display individual data points along with the interquartile range (IQR) and the median (dashed lines) for each group. Statistical comparisons were performed using the Kruskal–Wallis test followed by Dunn’s multiple comparison test; * *p* < 0.05, ** *p* < 0.01, and *** *p* < 0.001. (**F**) Variable importance plot generated using a RF model to distinguish between active and inactive phases of SLE. The red dashed line at zero indicates the threshold separating biomarkers with positive importance from those with negligible or negative contributions. Corresponding summary statistics (median, Q1–Q3) are presented in Table 2 and Table 3.

In contrast, despite not differing from the healthy controls when analyzed as a combined SLE group, the perforin and granulysin levels were consistently elevated in the majority of aSLE patients compared with the levels in the iSLE patients (Table 3; Figure 1A,B).

The levels of sFas and sFas-L were increased in the iSLE patients compared with the HDs (Table 2 and Table 3; Figure 1C,D). However, only the sFas levels were higher in the aSLE patients than in the HDs (Table 2 and Table 3; Figure 1C). On the other hand, the TNF-α levels were predominantly elevated in the iSLE patients compared with the HDs (Table 2 and Table 3; Figure 1E). These findings highlight specific biomarker variations associated with the clinical phases of SLE. In general, we observed a tendency toward elevated levels of specific cytotoxic markers in the aSLE patients (Table 3).

### 3.4. Important Serum Biomarkers Differentiating SLE Activity Phases

Furthermore, we tested whether biomarker quantification could aid in distinguishing between different levels of SLE activity. We identified key biomarkers associated with disease activity, as determined by the random forest (RF) algorithm (Figure 1F).

The RF model predicted the active or inactive SLE phase through analysis of biomarkers, including cytokines, cytotoxic molecules, and traditional markers such as C3, C4, and anti-dsDNA (SLEDAI-2K parameters). The model uses decision trees to optimize data classification on the basis of variable importance, thereby identifying key biomarkers for distinguishing SLE activity. This highlights its potential as a complementary diagnostic tool for managing SLE patients.

Among the analyzed parameters, perforin, granulysin, and granzyme-B were identified as the most influential factors, with the highest positive importance scores. These biomarkers play critical roles in the immunopathogenesis of SLE, and they appear to contribute significantly to the differentiation between the iSLE and aSLE groups. Another important parameter was Fas-L, which can promote the deregulation of apoptosis in patients [19]. On the other hand, parameters such as IFN-γ, IL-6, and IL-4 presented negative importance scores, suggesting their inverse contributions to the accuracy of the model’s predictive magnitude. Notably, conventional and clinical parameters of inflammation, such as C3, C4, and anti-dsDNA, which are commonly used to monitor SLE activity, were insufficient for accurate detection of the disease state in this model. This highlights the potential utility of incorporating measurements of cytokines, such as cytotoxic molecules, as additional biomarkers to improve disease prognosis and activity monitoring.

### 3.5. Cytotoxic Serum Markers Correlate with Disease Activity

To investigate the potential roles of the analyzed molecules in disease activity in SLE patients, we performed the Spearman correlation analysis between the serum levels of cytokines and cytotoxic molecules and the SLEDAI-2K score, a widely used clinical index for assessing disease activity. Both the active and inactive patients were included to evaluate the associations of these molecules with clinical disease activity.

Our analysis revealed significant positive correlations for perforin (r = 0.41, *p* = 0.002) and granulysin (r = 0.32, *p* = 0.017) (Supplementary Figure S2), suggesting that higher levels of these proteins are associated with increased disease activity. Interestingly, despite finding no significant differences in IL-6 concentrations between the patients with active and inactive SLE, we observed a significant positive correlation between IL-6 levels and disease activity (r = 0.33, *p* = 0.012) (Supplementary Figure S3). In contrast, the levels of IL-2, IL-4, IL-10, IFN-γ, IL-17A, TNF-α, granzyme-A, granzyme-B, sFas, and sFas-L were not significantly correlated (*p >* 0.05) (Supplementary Figures S2 and S3). To complement these findings with traditional clinical biomarkers, we also assessed complement factors C3 and C4 and anti-dsDNA antibodies. As expected, C3 (r = −0.34, *p* =0.009) and C4 (r = −0.49, *p* < 0.001) levels were negatively correlated with SLEDAI-2K, consistent with complement consumption during active disease. Conversely, anti-dsDNA antibody levels showed a positive correlation with disease activity (r = 0.26, *p* = 0.054), although this did not reach conventional statistical significance (Supplementary Figure S4) [20].

These correlations are essential for understanding immunological processes in the context of SLE. Notably, perforin and granulysin emerged as promising cytotoxic biomarkers, which may complement conventional parameters, such as C3, C4, and anti-dsDNA. This suggests that the analyzed molecules could serve as tools for monitoring disease activity, potentially providing additional precision in the clinical assessment in SLE patients.

### 3.6. CD8^+^ Cells Drive Distinct Immune Patterns in Patients with Active and Inactive SLE

Given the prominence of cytotoxic molecules observed in SLE patients, we sought to investigate the role of CD8^+^ cells as an important source of these cytotoxic biomarkers, which may contribute to the immunopathogenesis of the disease.

**Figure 2:**
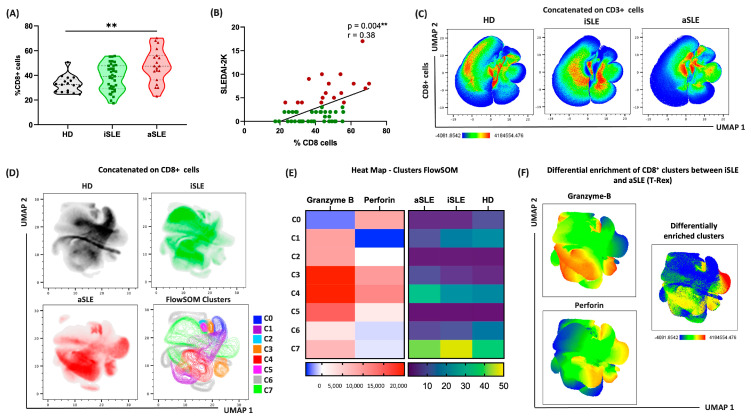
CD8^+^ cells are related to cytotoxic enrichment in active SLE. (**A**) Violin plots showing peripheral CD8^+^ cell frequencies in healthy donors (HD; *n* = 17), inactive SLE (iSLE; *n* = 36) and active SLE (aSLE; *n* = 19) patients. (**B**) Positive correlation between CD8^+^ cell percentage and SLE disease activity (SLEDAI-2K) in patients (*n* = 55). Red dots represent active SLE (aSLE) patients; green dots represent inactive SLE (iSLE) patients. (**C**) UMAP of CD8^+^ cell distributions across HDs, iSLE, and aSLE. (**D**) UMAP of CD8^+^ cells colored by group and FlowSOM clustering (C0–C7). (**E**) Heatmap showing expression of granzyme B and perforin across FlowSOM clusters and their sample enrichment by disease group. (**F**) T-Rex analysis identifying differentially enriched clusters between iSLE and aSLE patients based on granzyme B and perforin expression. Violin plots show interquartile range (IQR) and median as dotted lines. Statistical comparisons were performed using the Kruskal–Wallis test, followed by Dunn’s multiple comparison test. ** *p* < 0.01.

We isolated PBMCs and quantified the percentage of CD8^+^ cells in peripheral blood. Our analysis revealed an increase in the frequency of CD8^+^ cells in the aSLE patients compared with the HDs (*p* < 0.001; Figure 2A). Moreover, CD8^+^ cell frequency positively correlated with disease activity measured by the SLEDAI-2K score (r = 0.38; *p*= 0.004; Figure 2B).

To explore phenotypic differences, we performed unsupervised dimensionality reduction via uniform manifold approximation and projection (UMAP), which revealed distinct CD8^+^ cell distributions across the HDs, iSLE, and aSLE groups (Figure 2C). Subsequent clustering using the FlowSOM algorithm on CD8^+^ cells identified eight distinct clusters (C0–C7), based on granzyme-B and perforin expression levels (Figure 2D). Cytotoxic clusters were differentially enriched among disease states, with higher expression levels observed in the aSLE patients (Figure 2E).

Finally, to further dissect enrichment patterns between the iSLE and aSLE patients, we employed the T-Rex algorithm, which confirmed the presence of differentially enriched clusters within CD8^+^ cells based on granzyme-B and perforin levels (Figure 2F).

Altogether, our data reveal increased frequencies of cytotoxic CD8^+^ cells in aSLE patients and demonstrate disease-specific phenotypic profiles, suggesting a potential role for these cells in SLE pathogenesis.

### 3.7. Cytotoxic CD8^+^ Cells Are Predominant in Patients with Active Disease

**Figure 3:**
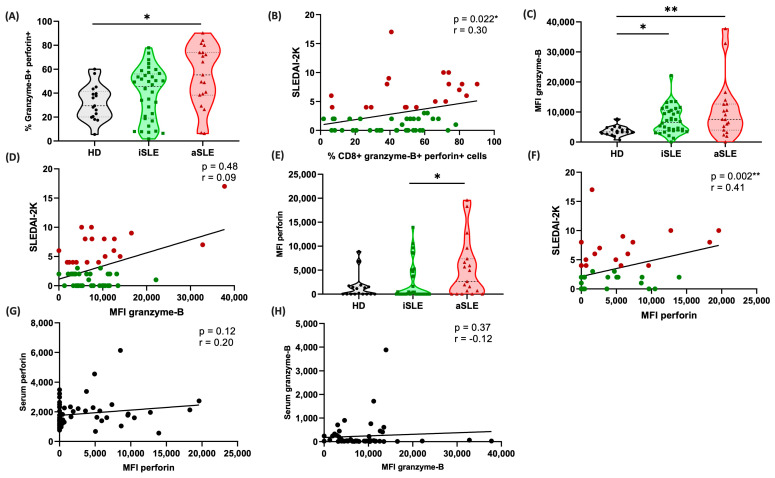
Predominance of cytotoxic CD8^+^ cells in patients with active SLE. (**A**) Violin plots showing the percentages of peripheral cytotoxic T cells (CD8^+^granzyme-B^+^perforin^+^) in the healthy donors (HD; *n* = 17), inactive SLE (iSLE; *n* = 36) and active SLE (aSLE; *n* = 19) patients. (**B**) Positive correlation between the percentage of cytotoxic cells (n = 55) and disease activity, as measured by the SLEDAI-2K score. (**C**) Median fluorescence intensity (MFI) of granzyme-B, showing differences between the groups. (**D**) Lack of correlation between the granzyme-B MFI (*n* = 55) and disease activity (SLEDAI-2K score). (**E**) MFI of perforin, which was significantly different between the groups. (**F**) Positive correlation between the perforin MFI (*n* = 55) and disease activity (SLEDAI-2K score). Red dots represent active SLE (aSLE) patients; green dots represent inactive SLE (iSLE) patients. (**G**,**H**) Evaluation of the serum levels of granzyme-B and perforin, which were not significantly correlated with their respective cellular MFI levels. The violin plots display the interquartile range (IQR) and median as dashed lines. Statistical comparisons were performed using the Kruskal–Wallis test, followed by Dunn’s multiple comparison test. * *p* < 0.05, ** *p* < 0.01.

Once we identified the potential cytotoxic patterns of CD8^+^ cells, we focused on evaluating the subset of cytotoxic cells defined as CD8^+^granzyme-B^+^perforin^+^ cells to determine their contribution to the pathogenesis of SLE. Our data revealed a significant increase in the frequency of these cells in patients with aSLE compared with the HD group (*p* = 0.01; Figure 3A). Furthermore, this frequency positively correlated with SLE disease activity, as measured using the SLEDAI-2K score (r *=* 0.30, *p* = 0.022; Figure 3B). This analysis was performed including both iSLE and aSLE patients to capture the overall trend of cytotoxic cell frequency in relation to disease activity. While separate analyses could yield different correlation values, our goal was to assess the general association across the SLE spectrum.

In addition to the frequency of cells, we evaluated the median fluorescence intensities (MFIs) of both granzyme-B and perforin. The MFI of granzyme-B was significantly greater in the aSLE patients than in the HDs (*p* = 0.003). Interestingly, the iSLE patients also presented increased granzyme-B levels compared with HDs (*p* = 0.01; Figure 3C). However, when the MFIs were compared between the iSLE and aSLE patients, no significant difference was observed. Furthermore, no significant correlation was found between the granzyme-B MFI and the SLEDAI-2K score (r = 0.09, *p* = 0.48; Figure 3D), indicating that its level may not be directly associated with disease activity.

On the other hand, the MFIs of perforin were significantly different between the aSLE and iSLE patients (*p* = 0.04; Figure 3E). Importantly, the perforin MFI was positively correlated with the SLEDAI-2K score (r = 0.41, *p* = 0.002; Figure 3F), suggesting an association of perforin with disease activity in contrast to granzyme-B.

Cytotoxic T lymphocytes (CTLs) and natural killer (NK) cells are known to mediate immune responses through the secretion of cytolytic granules, such as perforins, granzymes, and granulysin [21,22]. In this context, we evaluated whether the serum levels of granzyme-B and perforin cytotoxic molecules correlated with their MFI values obtained via flow cytometry. However, no significant correlations were detected between the serum levels of these molecules and their cellular MFI values (Figure 3G,H). Therefore, these data suggest that cytotoxic CD8^+^ cells may not be the only source of these molecules in SLE patients.

### 3.8. Cytotoxic Patterns of Cells and Proteins Differ Across the Phases of SLE Activity

**Figure 4:**
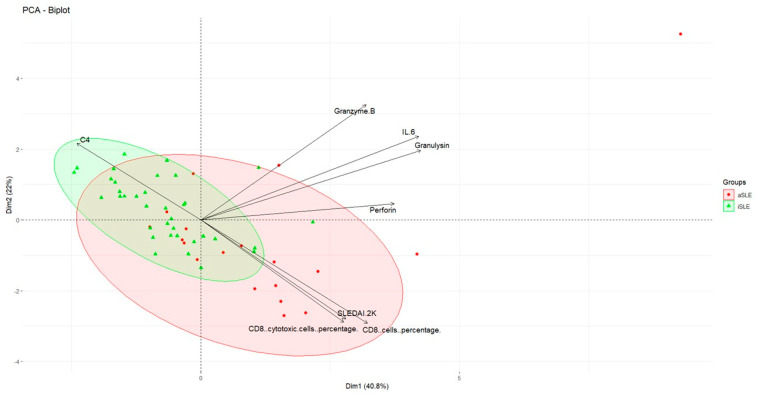
Cytotoxic and immune parameters associated with SLE activity. The PCA biplot illustrates the distribution of inactive SLE patients (iSLE, *n* = 36; green triangles) and active SLE patients (aSLE, *n* = 19; red circles). The confidence ellipses identify the two groups. The arrows indicate the contributions of specific variables (C4, IL-6, granzyme-B, granulysin, perforin, SLEDAI-2K score, CD8^+^ cell percentage, and CD8^+^ cytotoxic cell percentage) to the differentiation between active and inactive disease states along the principal components.

After identifying potential cytotoxic patterns of CD8^+^ cells, we performed a PCA to determine whether the set of studied parameters could provide information on the disease status of SLE patients in the active and inactive phases. The key variables previously identified as relevant to cytotoxicity and SLE activity were selected, as they provided the most meaningful separation between the groups while minimizing potential noise from less informative markers (Figure 4). The PCA biplot revealed a partial separation between the two groups in the bidimensional space generated by the first two principal components, Dim1 (40.8%) and Dim2 (22%), which together explained 62.8% of the total variance.

Although some overlap was observed between the samples, the patients with aSLE tended to cluster toward the right side of the plot, where variables such as perforin, granulysin, granzyme-B, and IL-6 had the greatest contributions to Dim1, thus driving the separation of the aSLE patients. Similarly, the SLEDAI-2K score, along with the percentages of total CD8^+^ and cytotoxic CD8^+^ cells reflected disease activity. In contrast, the C4 levels in the iSLE patients were closer to the origin and clustered toward the left, suggesting a lower expression of this marker and a shift away from Dim2 in inactive disease (Figure 4). These directional variations reflect the composite nature of the PCs, where each marker contributes with a specific weight to the overall variance structure.

To further evaluate the discriminatory power of these markers, we conducted ROC curve analyses for individual biomarkers between the two groups of SLE patients (iSLE and aSLE) (Supplementary Figure S5). While perforin and granulysin showed moderate performance individually (AUCs 0.77 and 0.73, respectively), these results suggest that no single marker was sufficient to fully discriminate against disease states.

**Figure 5:**
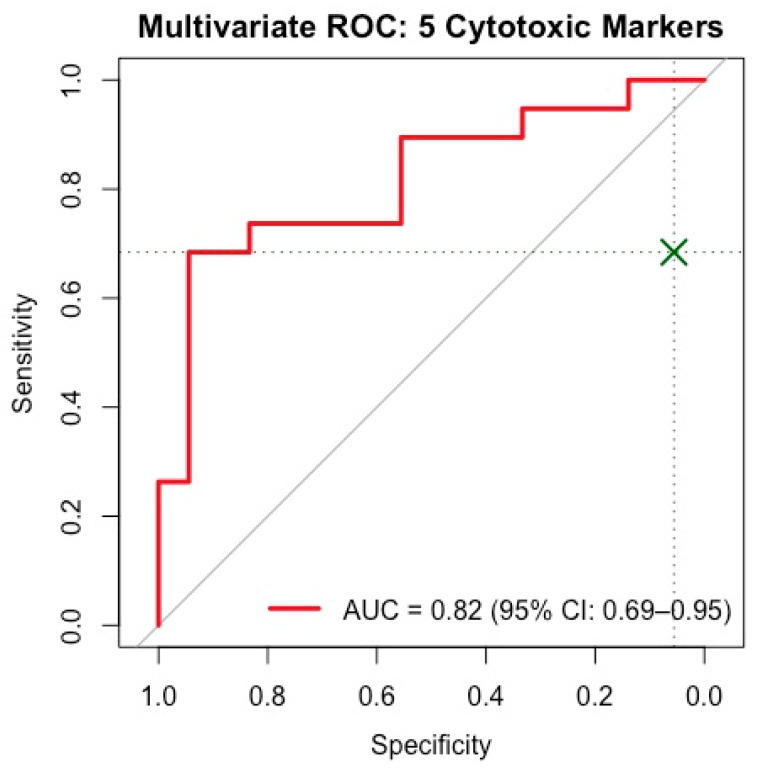
ROC curve derived from a multivariate logistic regression model including perforin, granulysin, granzyme-B, %CD8^+^ T cells and % CD8^+^ cytotoxic T cells. The model achieved an AUC of 0.82 (95% CI: 0.69–0.95) with an optimal cutoff of 0.45 (Youden’s index), yielding 68% sensitivity and 94% specificity. The green “X” and dotted lines indicate the optimal cutoff point according to Youden’s index. Among the variables, only granulysin was statistically significant (*p* = 0.016).

To address this, we performed multivariate logistic regression analyses. A simplified 3-marker model (perforin, granulysin, and CD8^+^ cytotoxicity) yielded an AUC of 0.77 (95% CI: 0.62–0.92), with 68% sensitivity and 86% specificity at an optimal cutoff (Youden’s index = 0.35) (Supplementary Figure S6). To enhance classification, we implemented a 5-marker model, including granzyme-B and %CD8^+^ cells. This model achieved an AUC of 0.82 (95% CI: 0.69–0.95), with improved specificity (94%) and the same sensitivity (68%) at the optimal cutoff point (Youden’s index = 0.45) (Figure 5). Although only granulysin reached statistical significance (*p* = 0.016), the combined model demonstrated superior discriminative capacity. The odds ratios for all variables are summarized in Supplementary Table S2.

Together, these findings suggest that a composite cytotoxic signature provides superior discriminative power for distinguishing active from inactive SLE, reinforcing the relevance of CD8^+^ T-cell-related pathways in the pathogenesis and stratification of disease activity.

## 4. Discussion

SLE is primarily mediated by B cells because of the presence of autoantibodies, which drive the development of autoimmunity [23]. While anti-dsDNA scoring is commonly used in clinical practice for diagnosing SLE [24], previous studies have emphasized the importance of serum cytotoxic biomarkers produced by T cells [14,25,26]. However, to the best of our knowledge, no prior study has proposed the combination of biomarkers that were analyzed in this study as a tool to differentiate between the active and inactive stages of SLE.

In this study, we evaluated disease activity phases by analyzing 13 molecules, including cytokines and cytotoxic molecules, in a cohort of 55 Mexican SLE patients, of whom 36 were in the inactive phase and 19 were in the active phase of the disease. Overall, we observed that elevated serum levels of cytotoxic molecules, combined with clinical and cellular immune response parameters, could improve the diagnostic accuracy and potentially contribute to better treatment approaches for SLE patients.

In line with previous studies [14,27], we observed elevated serum levels of TNF-α, sFas, and sFas-L (*p* = 0.009, *p* < 0.001, and *p* = 0.045, respectively) in the overall SLE group compared with HDs. Granzyme-B also exhibited a trend toward increased levels (*p* = 0.075), although this difference did not reach statistical significance, possibly due to treatment heterogeneity, disease stage, or interindividual variability. In contrast, other cytokines and cytotoxic molecules, such as IL-2, IL-4, IL-6, IL-10, IFN-γ, IL-17A, perforin, and granulysin, did not significantly differ between the SLE patients and HDs. This finding contrasts with previous reports [28,29] describing broader cytokine dysregulation in SLE. These discrepancies may be explained by differences in cohort characteristics, such as ethnicity, disease duration, or immunosuppressive treatment exposure.

In the comparisons between the SLE subgroups, the serum concentrations of perforin and granulysin were significantly elevated in the patients with aSLE compared to those with iSLE. Moreover, the sFas/sFas-L and TNF-α levels were elevated in the iSLE patients compared with those in the HDs, but only the sFas levels were increased in the aSLE patients compared with the HDs. Among the molecules, the RF model highlighted the greater importance of cytotoxic molecules, such as perforin, granulysin, and granzyme-B, in distinguishing between disease phases. Additionally, the serum perforin and granulysin levels correlated significantly with the SLEDAI-2K score, reinforcing their potential role as biomarkers of disease activity.

On the other hand, IL-6 promotes T follicular helper (Tfh) cell differentiation and the formation of spontaneous germinal centers (GCs), the site of autoantibody-producing B cells [9]. Although increased levels of this cytokine have also been reported in previous studies of SLE [7,30,31], this cohort did not show differences in the IL-6 levels between patients with active and inactive SLE. However, the IL-6 levels correlated with the SLEDAI-2K disease activity scores. This may reflect the clinical heterogeneity of SLE and the effects of treatment, which likely reduce IL-6 levels and group differences.

Importantly, many patients in this cohort were on corticosteroids, such as prednisone, which may have influenced their immune profiles, including the serum levels of cytokines and cytotoxic molecules. Corticosteroids are known to suppress inflammatory cytokines, such as IL-6, IFN-γ, and TNF-α [32], and can affect the functional state of T cells [33,34], such as Treg expansion [35] or CD8^+^ cell metabolism [36,37]. The variability in corticosteroid dosages may partly explain the lack of significant differences between groups, potentially masking stronger correlations with SLEDAI-2K scores. Moreover, corticosteroids can inhibit cytotoxic mediators, such as perforin and granzyme-B [38]; however, our findings suggest that perforin and granulysin may remain reliable biomarkers of disease activity even under corticosteroid therapy.

It is important to note that perforin and granulysin are also markers of NK cells, which can be affected by immunosuppressive treatments, such as azathioprine and mycophenolate [39,40]. While our study was not designed to specifically assess treatment effects, the presence of these cytotoxic markers in CD8^+^ T cells suggests an active role of these cells in SLE pathogenesis beyond NK cell contributions. Future studies controlling for treatment effects will be necessary to confirm these findings.

In the context of SLE, dysregulation of CD8^+^ cell function has been reported to contribute to immune-mediated tissue damage and disease progression [41]. In our study, we observed a significant increase in the percentage of CD8^+^ cells in patients with active SLE, which further correlated with disease activity, as measured by the SLEDAI-2K. This suggests that CD8^+^ cells promote cellular deregulation during the active phase of SLE.

Furthermore, our unsupervised flow cytometry analysis revealed distinctive clustering patterns among the HD, iSLE, and aSLE groups. This finding strengthens the notion that SLE activity is associated not only with quantitative changes but also with qualitative changes in the CD8^+^ T-cell compartment. Notably, we identified eight distinct CD8^+^ cell subpopulations characterized by granzyme-B and perforin expression, with a greater abundance of these clusters in SLE patients. Our results are consistent with the findings reported by Fogagnolo et al. (2014) [42], who demonstrated that in cutaneous lupus erythematosus, persistent inflammation was associated with the presence of cytotoxic granules, including granzymes, perforin, and granulysin. The predominant cell population producing these molecules may be CD8^+^ cells [42].

Mechanistically, perforin and granzyme-B have been shown to contribute to vascular injury by inducing apoptosis in endothelial cells [43,44], a process relevant to the vascular manifestations and tissue damage observed in SLE. These mechanisms may underlie the observed association between elevated serum perforin levels and disease activity in our cohort.

Importantly, both the percentage of granzyme-B^+^perforin^+^ CD8^+^ cells and the perforin MFI were significantly positively correlated with the SLEDAI-2K score. However, no significant positive correlations were detected between the perforin and granzyme-B MFIs and their serum levels. This may reflect the fact that serum concentrations indicate systemic processes rather than cell numbers or activity. Additionally, these molecules are known to be regulated by inhibitory proteins, which may diminish their detectable associations with the producing cells [45].

Similarly to our findings, Patrick Blanco et al. reported increased numbers of cytotoxic CD8^+^ cells (CD8^+^perforin^+^ and CD8^+^granzyme-B^+^ cells) in aSLE patients, along with a positive correlation with the SLEDAI scores [10]. These findings underscore the complexity of SLE immunopathogenesis and suggest that a combination of diverse biomarkers (cytotoxic molecules, genetic profiles, and immune checkpoints) could provide a more accurate evaluation of disease activity.

Previous studies have identified aberrant genetic signatures in CD8^+^ cells in SLE, highlighting the relevance of cytolytic granules to advanced lupus nephritis (LN) phenotypes. Additionally, the expression of coinhibitory receptors on CD8^+^ cells has been correlated with both disease activity and cumulative organ damage in SLE patients [46]. This raises the possibility that other CD8^+^ cell subpopulations contribute to disease pathogenesis, either through the secretion of additional cytotoxic molecules or by transitioning to a cellular exhausted state [47,48,49]. Together, these findings underscore the multifaceted role of CD8^+^ cells in SLE pathogenesis, which act as both drivers of inflammation and potential mediators of tissue injury.

However, a limitation of our study is the absence of markers used to define T-cell differentiation states, such as CD45RA, CD45RO, and CCR7. These markers allow for a more precise functional and developmental categorization of CD8^+^ T-cell subsets. While our cytometric analysis focused on cytotoxic molecule expression, integrating peripheral differentiation markers would have enriched the immunophenotypic resolution of the CD8^+^ compartment, especially since significant shifts have been demonstrated in T-cell memory subsets in lupus nephritis patients [50].

Furthermore, we did not perform advanced functional assays to evaluate the cytolytic or suppressive capacity of CD8^+^ T cells, such as ex vivo stimulation assays, intracellular cytokine staining, or degranulation analyses, which have been previously used to assess functional competence in SLE patients [51]. Incorporating these assays in future studies would be essential to validate whether the observed phenotypes correspond to functionally active or dysfunctional states of CD8^+^ T cells.

Regarding other limitations, the cross-sectional design of our study precludes the analysis of temporal changes in immune parameters during disease flares and remission. Although longitudinal studies would provide more robust insights into biomarker dynamics, they are often constrained by logistical challenges, such as difficulty recruiting patients during acute flares. Nevertheless, our findings provide a valuable snapshot of immune dysregulation associated with disease activity in SLE.

Finally, the relatively small sample size, particularly in the aSLE group, limits the statistical power of subgroup analyses. Although stratification by disease duration or severity might reveal additional subgroup-specific patterns, data on disease duration are not consistently available across all patients. Future studies involving larger and more comprehensively characterized cohorts will be essential to validate and extend our findings.

Despite this limitation, a multivariate logistic regression model incorporating perforin, granulysin, granzyme-B, %CD8^+^ T cells, and %CD8^+^ cytotoxic T cells yielded a good discriminatory performance (AUC = 0.82), with 94% specificity and 68% sensitivity at the optimal cutoff. This high specificity suggests that the composite cytotoxic signature could be particularly valuable for confirming disease activity in clinical settings. These findings also highlight the clinical and cellular variables that contributed to group separation in the PCA model.

In general, our findings demonstrate that cytotoxic molecules, such as perforin, granzyme-B and granulysin, along with other immunological markers, such as C4 and IL-6, could be pivotal in characterizing the disease phases of SLE. Additionally, our findings add to the growing body of evidence supporting the relevance of CD8^+^ cell dysfunction to SLE. This combination provides a more integrated view of immune dysregulation that occurs during active diseases. By integrating clinical parameters with cellular and molecular data, we provide a multifaceted perspective on immune dysregulation during the active phase of this disease. The combination of biomarkers proposed in this study offers a novel approach to the stratification of patients with SLE, laying the groundwork for identifying potential therapeutic targets and improving patient care.

## Figures and Tables

**Table 1:** Demographic and clinical characteristics of study subjects.

	HD	SLE	iSLE	aSLE
Total *n*	17	55	36	19
Female	14 (82%)	46 (83%)	30 (83%)	16 (84%)
Age, years	26 (25–53)	36 (28–46)	36 (30–44)	34 (28–51)
Body Mass Index	-	25.3 (21.9–29.5)	25.7 (22.0–30.4)	24.6 (19.5–27.3)
Systolic Blood Pressure	-	119 (109–129)	120 (108–124)	115 (109–131)
Diastolic Blood Pressure	-	69 (61–76)	65 (61–72)	71 (62–77)
Comorbidities				
Diabetes	0	4 (7.3%)	2 (5.5%)	2 (10.5%)
Hypertension	0	16 (29.1%)	10 (27%)	6 (31.5%)
Hypothyroidism	0	9 (16.4%)	5 (13.8%)	4 (21.0%)
Chronic Kidney Disease	0	9 (16.4%)	7 (19.4%)	2 (10.5%)
Dyslipidemia	0	1 (1.8%)	1 (2.7%)	0 (0%)
Myocardial infarction	0	0 (0%)	0 (0%)	0 (0%)
Stroke	0	2 (3.6%)	1 (2.7%)	1 (5.2%)
Lung disease	0	3 (5.5%)	1 (2.7%)	2 (10.5%)
SLE duration, years	NA	7 (3–18)	6 (3–14.7)	15 (5–24)
SLEDAI 2K	NA		0 (0–2)	6 (4–8)
Neurological ^a^	NA	0	0	0
Vascular	NA	1	0	1
Articular	NA	1	0	1
Muscular	NA	0	0	0
Renal ^b^	NA	19	0	19
Cutaneous ^c^	NA	1	0	1
Serositis	NA	0	0	0
Biochemical ^d^	NA	32	15	17
Anti-dsDNA	NA	30.2 (10.3, 124.4)	29.3 (8.8, 123.8)	36.5 (14.0, 321.7)
C3	NA	98.1 (73.0, 109.0)	100.1 (79.5, 108.8)	81.7 (70.4, 114.0)
C4	NA	14.0 (9.1, 20.0)	15.0 (9.7, 20.6)	10.5 (4.4, 15.5)
Hematological ^e^	NA	9	4	5
Constitutional (Fever)	NA	0	0	0
Treatments				
Corticosteroids	NA	21 (38.2%)	9 (25.0%)	12 (63.1%)
Antimalarials	NA	36 (65.4%)	24 (66.6%)	12 (63.1%)
Immunosuppressants				
Mycophenolate	NA	41 (74.5%)	25 (69.4%)	16 (84.2%)
Azathioprine	NA	9 (16.4%)	7 (19.44%)	2 (10.5%)
Rituximab	NA	1 (1.8%)	1 (2.7%)	0 (0%)
Other ^f^	NA	10 (18.8%)	7 (19.4%)	3 (15.7%)

Abbreviations: HD: Healthy donor; iSLE: Patient with inactive lupus; aSLE: Patient with active lupus. Quantitative data are expressed as median (interquartile range), and qualitative data as *n* (%). SLEDAI-2K: Systemic Lupus Erythematosus Disease Activity Index 2000. ^a^ Includes recent-onset seizure, psychosis, organic brain syndrome, visual disturbances, new-onset neuropathy, lupus headache, and new-onset stroke. ^b^ Includes urinary casts, hematuria, proteinuria, and pyuria. ^c^ Includes inflammatory rash, alopecia, and mucosal ulcers. ^d^ Includes low complement and high anti-dsDNA antibody levels. ^e^ Includes thrombocytopenia and leukopenia. ^f^ Includes tacrolimus, methotrexate, cyclophosphamide, and cyclosporine. NA: Not applicable.

**Table 2:** Comparison of serum cytokine and cytotoxic molecule levels between healthy donors (HD) and SLE patients.

Parameters	HD (Median; Q1–Q3)	SLE (Median; Q1–Q3)	*p*-Value
IL-2	47.87 (36.34–64.23)	46.93 (36.34–89.13)	0.75
IL-4	9.36 (4.76–13.19)	8.95 (5.92–21.59)	0.45
IL-6	17.57 (14.06–28.74)	19.89 (14.48–30.67)	0.37
IL-10	5.68 (5.68–11.60)	7.39 (5.68–15.77)	0.29
IFN-γ	39.10 (24.47–97.56)	52.07 (35.01–123.6)	0.18
IL-17A	1.64 (1.37–3.09)	2.14 (1.40–7.17)	0.21
TNF-α	8.13 (5.59–12.98)	14.04 (7.21–31.19)	0.009 **
Granzyme-A	65.62 (41.95–114.7)	91.16 (49.98–202.1)	0.1
Granzyme-B	22.16 (17.49–40.19)	35.03 (21.90–238.1)	0.075
sFas	54.12 (28.38–75.57)	199 (101.5–491.1)	<0.001 ***
sFas-L	60.45 (51.31–67.78)	44.19 (30.54–66.23)	0.045 *
Perforin	1757 (1538–2297)	1628 (1214–2283)	0.37
Granulysin	1044 (944.7–1398)	1171 (672–1794)	0.91

Values are expressed in pg/mL as medians with first (Q1) and third (Q3) quartiles. Statistical comparisons between HD and SLE patients were performed using the nonparametric Mann–Whitney U test. * *p* < 0.05, ** *p* < 0.01, *** *p* < 0.001.

**Table 3:** Comparison of serum cytokine and cytotoxic molecule levels between inactive (iSLE) and active (aSLE) SLE patients.

Parameters	iSLE (Median; Q1–Q3)	aSLE (Median; Q1–Q3)	*p*-Value
IL-2	47.70 (36.34–85.72)	42.68 (36.34–115.9)	0.45
IL-4	8.95 (5.92–21.59)	10.01 (4.50–59.06)	0.54
IL-6	18.70 (14.06–26.48)	24.26 (15.44–33.73)	0.08
IL-10	6.02 (5.68–13.02)	9.75 (5.68–17.51)	0.11
IFN-γ	51.61 (35.04–120.1)	55.78 (35.01–150.7)	0.74
IL-17A	2.5 (1.47–7.07)	1.98 (1.3–7.17)	0.46
TNF-α	15.13 (8.75–31.19)	12.58 (5.44–73.87)	0.35
Granzyme-A	84.02 (50.41–185.3)	95.13 (46.67–416.8)	0.32
Granzyme-B	35.47 (22.95–259.1)	28.17 (13.08–88.15)	0.38
sFas	202.9 (93.89–492.8)	175.4 (104.5–491.1)	0.66
sFas-L	37.15 (21.68–57.05)	49.22 (36.48–74.09)	0.03 *
Perforin	1444 (1092–1849)	2219 (1767–2491)	<0.001 ***
Granulysin	963.6 (550.4–1445)	1347 (1155–2576)	0.005 **

Values are expressed in pg/mL as medians with first (Q1) and third (Q3) quartiles. Statistical comparisons between iSLE and aSLE groups were performed using the nonparametric Mann–Whitney U test. * *p* < 0.05, ** *p* < 0.01, *** *p* < 0.001.

## Data Availability

Data are available upon request to the corresponding author (akarina_chavez@yahoo.com.mx).

## Supplementary Materials

The following supporting information can be downloaded at: https://www.mdpi.com/article/10.3390/biomedicines13071559/s1, Figure S1: Levels of cytokines and cytotoxic molecules in patients with SLE; Figure S2: Correlation of cytotoxic molecule levels with disease activity in SLE patients; Figure S3: Correlation of proinflammatory cytokine levels with disease activity in SLE patients; Figure S4: Correlation of clinical parameters with disease activity in SLE patients; Figure S5: ROC curves to discriminate active versus inactive SLE based on cytotoxic biomarkers; Figure S6: Multivariate ROC curve based on a logistic regression model including Perforin, Granulysin, and CD8^+^ cytotoxicity; Table S1: Assay sensitivity of the LEGENDplex™ Human CD8/NK Panel in serum samples; Table S2: Multivariate logistic regression analysis including five cytotoxic markers to classify active (aSLE) versus inactive SLE (iSLE).

## Abbreviations

The following abbreviations are used in this manuscript:

SLE	Systemic Lupus Erythematosus
iSLE	Inactive Systemic Lus Erythematosus
aSLE	Active Systemic Lupus Erythematosus
SLEDAI-2K	Systemic Lupus Erythematosus Disease Activity Index 2000
HD	Healthy Donors
PBMCs	Peripheral blood mononuclear cells
IL	Interleukin
TNF-α	Tumor necrosis factor-alpha
IFN-γ	Interferon gamma
PCA	Principal component analysis
IQR	Interquartile range
