# Supplementary material for: Cytotoxic Molecules as Potential Biomarkers for Active and Inactive Systemic Lupus Erythematosus
Source: Biomedicines. 2025 Jun 25;13(7):1559. doi: 10.3390/biomedicines13071559 (PMC12292586; doi:10.3390/biomedicines13071559)
Supplement: Supplementary file 1 [file biomedicines-13-01559-s001.zip › biomedicines-3631717-supplementary.pdf]

Supplementary Figure S1.

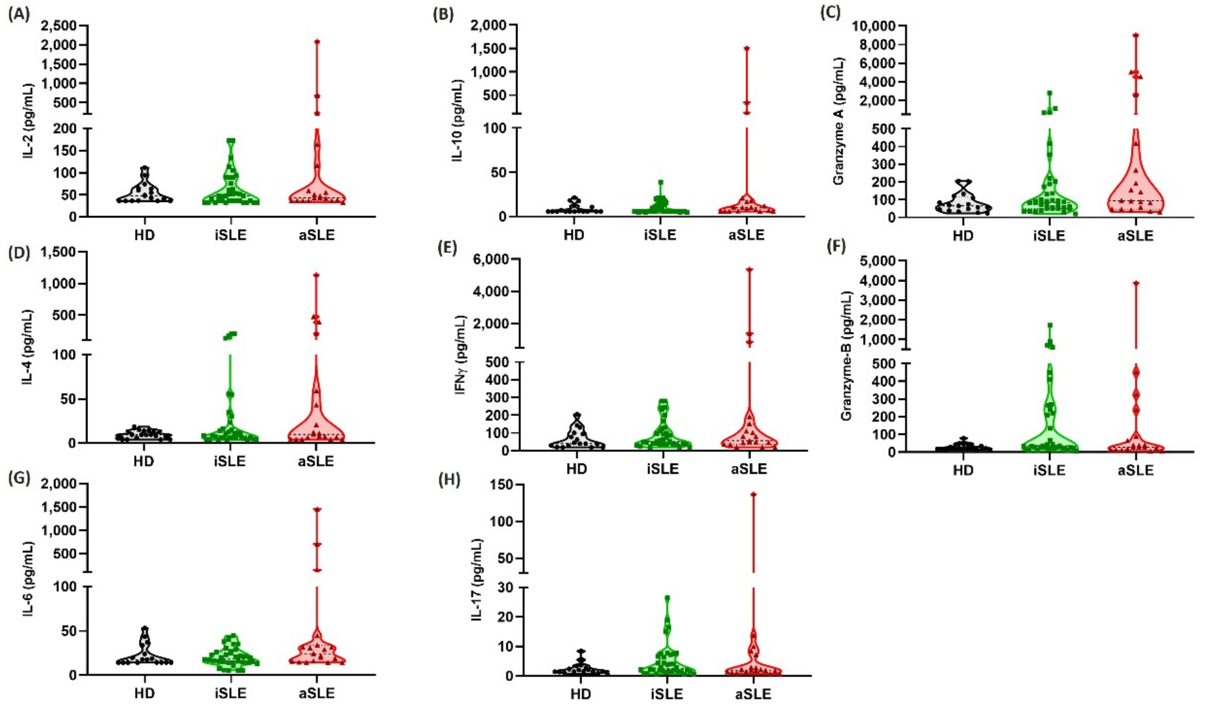

Supplementary Figure S1. Levels of cytokines and cytotoxic molecules in patients with SLE.

Panels A to H show nonsignificant differences in the levels of cytokines and cytotoxic molecules between patients with iSLE, aSLE and healthy donors (HDs). The violin plots display individual data points, along with median, minimum, and maximum values, of the analyzed proteins in iSLE patients (n = 36), aSLE patients (n = 19), and HDs (n = 17). Statistical comparisons were performed using the Kruskal–Wallis test, followed by Dunn's multiple comparison test. Note: All data points included in the analysis were within the quantifiable range of the assay. While some values were close to the lower limit of detection, they remained above the threshold defined by the LEGENDplex™ CD8/NK Panel for serum samples. No samples were excluded due to technical limitations or undetectable concentrations.

Supplementary Figure S2.

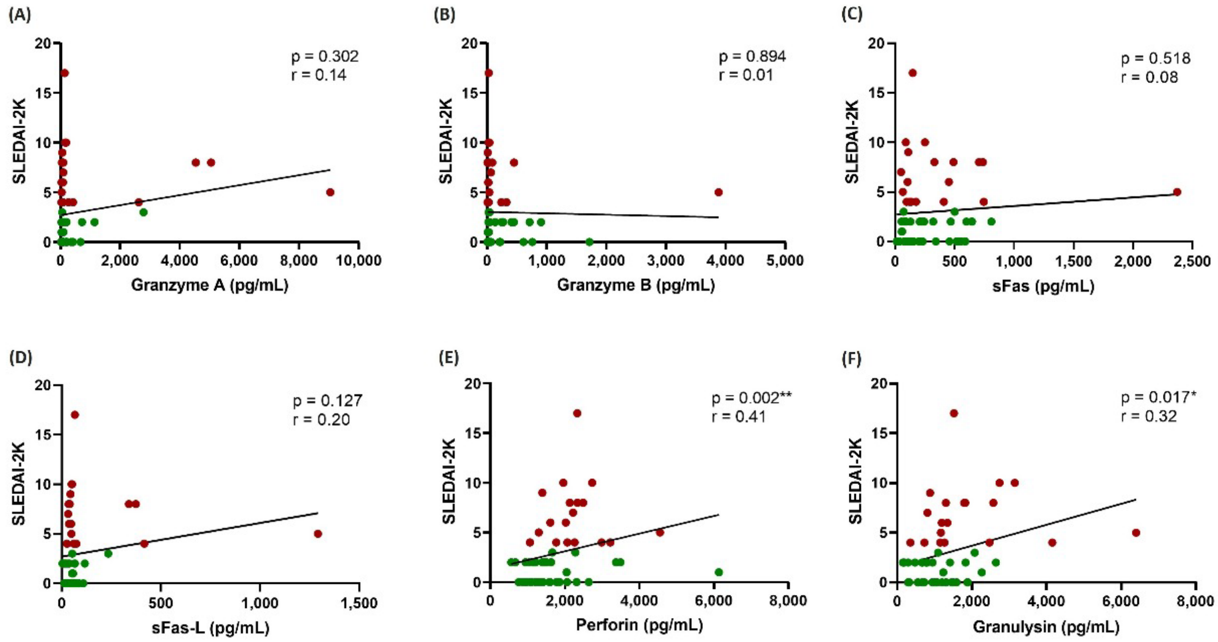

Supplementary Figure S2. Correlation of cytotoxic molecule levels with disease activity in SLE patients.

Panels A to F display the correlations between serum concentrations of cytotoxic molecules and SLEDAI-2K scores in patients with SLE. Patients with iSLE are represented in green, whereas patients with aSLE are shown in red. Correlation analyses were performed using Spearman's rank correlation test; p values < 0.05 were considered statistically significant. Note: All data points included in the analysis were within the quantifiable range of the assay. While some values were close to the lower limit of detection, they remained above the threshold defined by the LEGENDplex™ CD8/NK Panel for serum samples. No samples were excluded due to technical limitations or undetectable concentrations.

Supplementary Figure S3.

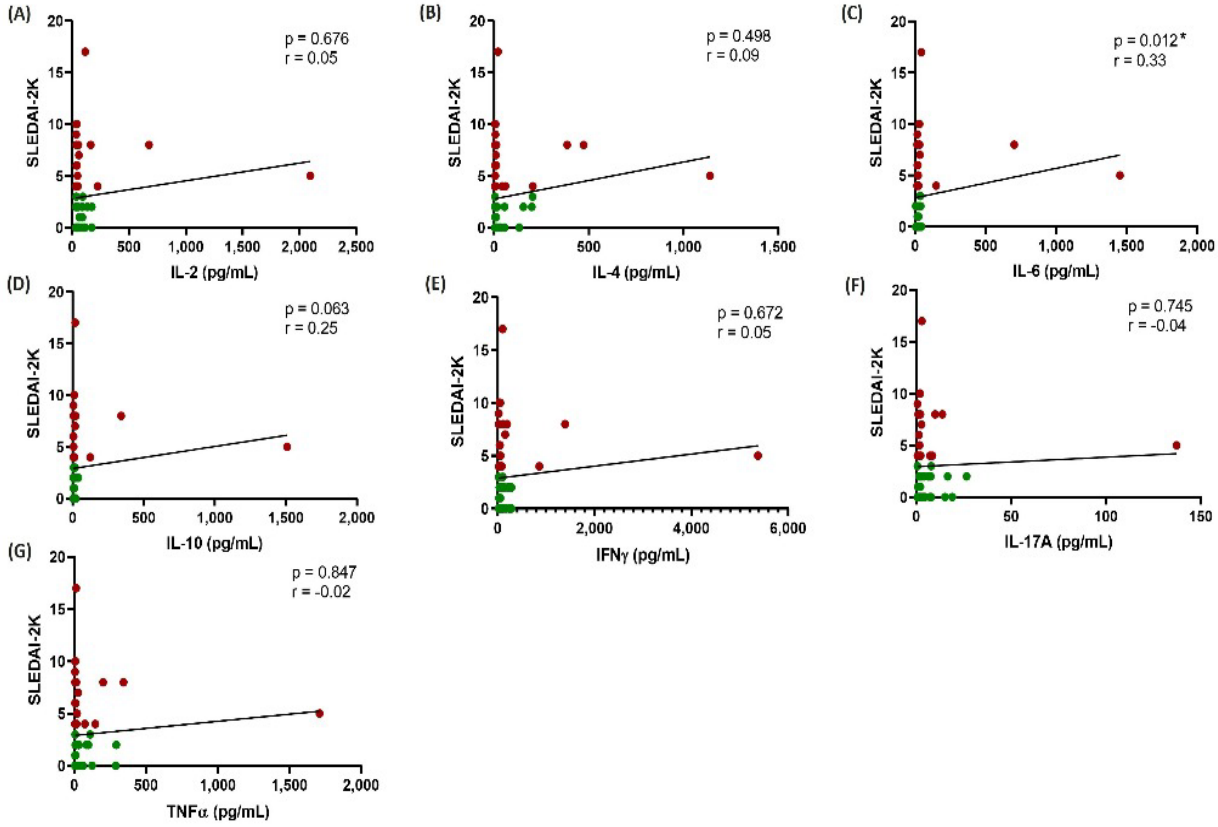

Supplementary Figure S3. Correlation of proinflammatory cytokine levels with disease activity in SLE patients.

Panels A to G show the correlations between serum concentrations of proinflammatory cytokines and SLEDAI-2K scores in patients with SLE. Patients with iSLE are represented in green, whereas patients with aSLE are shown in red. Correlation analyses were performed using Spearman's rank correlation test; *p* values < 0.05 were considered statistically significant. Note: All data points included in the analysis were within the quantifiable range of the assay. While some values were close to the lower limit of detection, they remained above the threshold defined by the LEGENDplex™ CD8/NK Panel for serum samples. No samples were excluded due to technical limitations or undetectable concentrations.

Supplementary Figure S4.

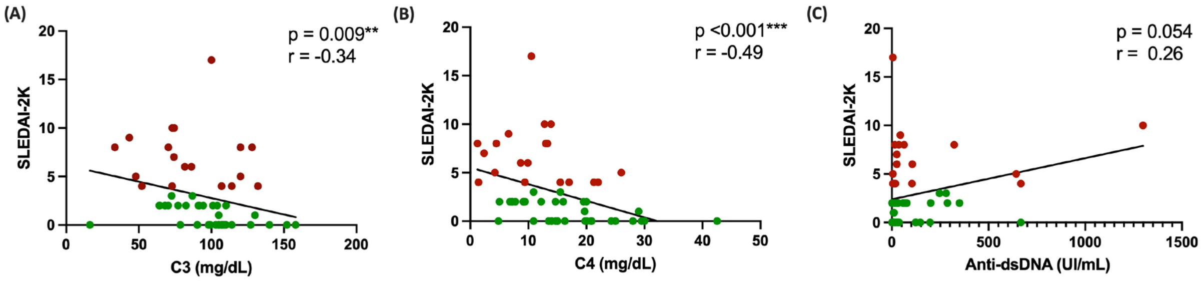

Supplementary Figure S4. Correlation of clinical parameters with disease activity in SLE patients.

Panels A to C display the correlations between SLEDAI-2K scores and serum concentrations of complement components (C3 and C4) as well as anti-dsDNA antibody levels. Patients with inactive SLE (iSLE) are shown in green, whereas those with active SLE (aSLE) are shown in red. Correlation analyses were performed using Spearman's rank correlation test, and p values < 0.05 were considered statistically significant. No samples were excluded.

Supplementary Figure S5.

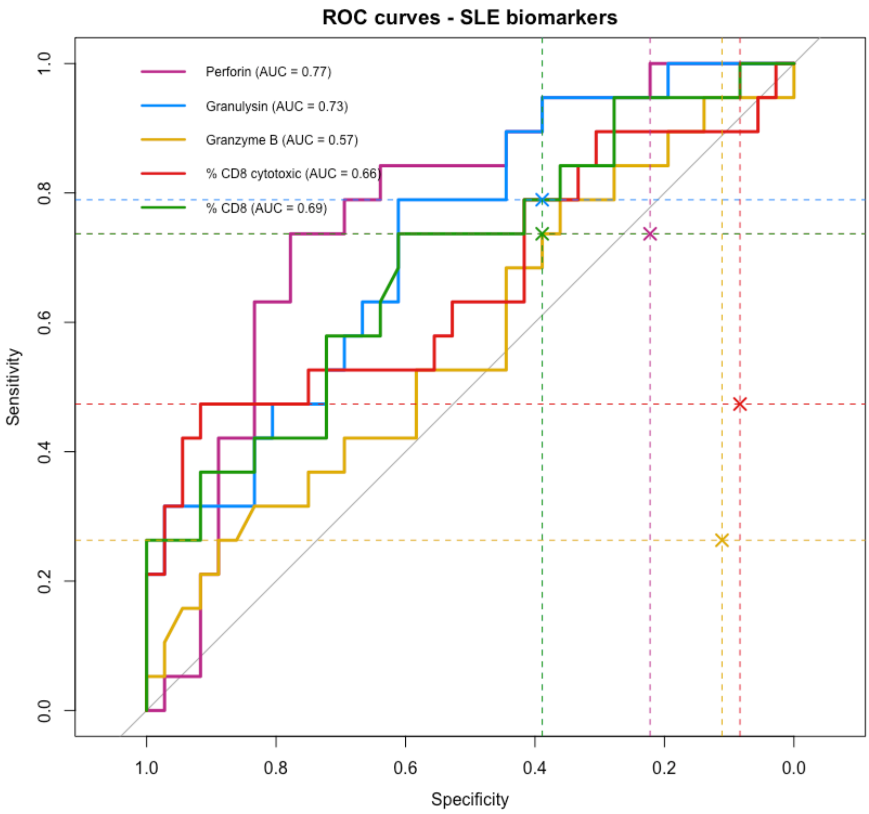

Supplementary Figure S5. ROC curves to discriminate active versus inactive SLE based on cytotoxic biomarkers.

AUC values with 95% confidence intervals and optimal cut-off points (Youden Index) are shown: Perforin (AUC = 0.77; 95% CI: 0.63–0.90; cut-off = 1907.79), Granulysin (AUC = 0.73; 95% CI: 0.59–0.87; cut-off = 1125.26), Granzyme B (AUC = 0.57; 95% CI: 0.41–0.74; cut-off = 15.37), % CD8 cytotoxic (AUC = 0.66; 95% CI: 0.49–0.83; cut-off = 65.40), and % CD8 (AUC = 0.69; 95% CI: 0.54–0.84; cut-off = 43.20).

Supplementary Figure S6.

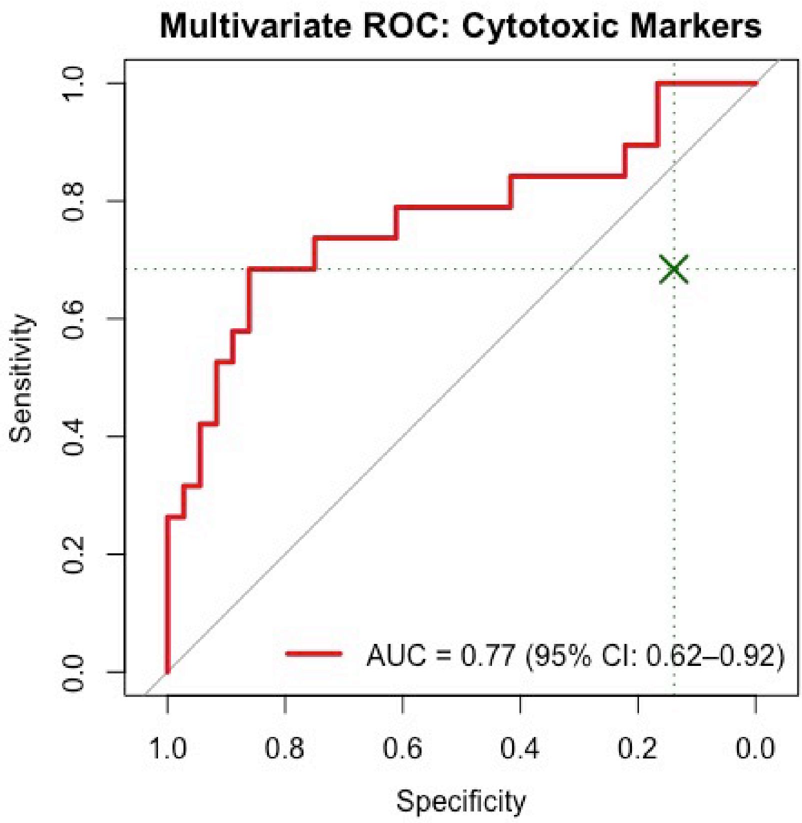

Supplementary Figure S6. Multivariate ROC curve based on a logistic regression model including Perforin, Granulysin, and CD8⁺ cytotoxicity.

This model yielded an AUC of 0.77 (95% CI: 0.62–0.92), with an optimal cutoff at 0.35 (Youden's index), achieving 68% sensitivity and 86% specificity. In the 3-marker logistic model, both granulysin (OR = 1.0014; 95% CI: 1.0004–1.0028; $p = 0.020$) and $CD8^+$ cytotoxicity (OR = 1.0333; 95% CI: 1.0036–1.0694; $p = 0.039$) were statistically significant predictors of active SLE.

Supplementary Table S1. Assay sensitivity of the LEGENDplex™ Human CD8/NK Panel in serum samples.

The limit of detection (LOD) in pg/mL was obtained from the manufacturer's technical datasheet. Values represent mean ± 2 SD for each analyte.

Analyte	LOD in Serum (pg/mL)
IL-17A	0.57 ± 0.80
IL-2	8.94 ± 10.59
IL-4	1.91 ± 3.19
IL-10	0.81 ± 0.71
IL-6	1.81 ± 2.21
TNF-α	1.58 ± 2.17
sFas	2.17 ± 1.07
sFasL	1.33 ± 2.67
IFN-γ	7.33 ± 10.50
Granzyme A	2.37 ± 2.66
Granzyme B	4.17 ± 3.81
Perforin	3.30 ± 5.13
Granulysin	11.47 ± 26.44

Supplementary Table S2. Multivariate logistic regression analysis including five cytotoxic markers to classify active (aSLE) versus inactive SLE (iSLE).

Variable	Odds Ratio	95% CI	p-Value
Perforin	0.9995	0.9985 – 1.0005	0.317
Granulysin	1.0016	1.0004 – 1.0031	0.016*
Granzyme B	0.9987	0.9968 – 1.0008	0.172
% CD8	1.0312	0.9679 – 1.1058	0.356
% CD8 cytotoxic	1.0233	0.9887 – 1.0630	0.204

Odds ratios (ORs) and 95% confidence intervals (CIs) were derived from a multivariate logistic regression model. Granulysin was the only marker that reached statistical significance ($p < 0.05$).
